# The Relationship between the Biofilm Genes and Antibiotic Resistance in *Stenotrophomonas maltophilia*

**DOI:** 10.1155/2023/8873948

**Published:** 2023-08-31

**Authors:** Fatemeh Sameni, Bahareh Hajikhani, Ali Hashemi, Parviz Owlia, Mohammad Niakan, Masoud Dadashi

**Affiliations:** ^1^Department of Microbiology, Faculty of Medicine, Shahed University, Tehran, Iran; ^2^Department of Microbiology, School of Medicine, Shahid Beheshti University of Medical Sciences, Tehran, Iran; ^3^Department of Microbiology, School of Medicine, Alborz University of Medical Sciences, Karaj, Iran; ^4^Non-Communicable Diseases Research Center, Alborz University of Medical Sciences, Karaj, Iran

## Abstract

**Objectives:**

Today, *Stenotrophomonas maltophilia* (*S. maltophilia*) is a major opportunistic pathogen among hospitalized or immunocompromised patients. Antibiotic-resistant clinical isolates are increasing in several parts of the world. Various antibiotic-resistance and biofilm-forming genes are identified in this bacterium. Its capacity to form biofilms is an important virulence factor that may impact antibiotic-resistance patterns. In the current study, we evaluated the biofilm-formation capacity, antibiotic-resistance profile, and prevalence of biofilm-forming genes as well as antibiotic resistance genes among *S. maltophilia* isolates.

**Materials and Methods:**

In this cross-sectional study, 94 clinical *S. maltophilia* isolates were recovered from four tertiary-care hospitals in Iran between 2021 and 2022. The presence of the selected antibiotic-resistance genes and biofilm-forming genes was examined by polymerase chain reaction (PCR). The ability of biofilm formation was examined by microtiter plate assay. The Kirby–Bauer disc diffusion method was used to evaluate the trimethoprim-sulfamethoxazole (TMP-SMX), levofloxacin, and minocycline resistance.

**Results:**

*S. maltophilia* is mainly isolated from bloodstream infections. Notably, 98.93% of isolates were biofilm producers, of which 19.35%, 60.22%, and 20.43% produced strong, moderate, and weak biofilm, respectively. The frequency of biofilm genes was 100%, 97.88%, 96.80%, and 75.53% for *spgM, rmlA, smf*-1, and *rpfF*, respectively. Isolates with the genotype of *smf*-1+/*rmlA*+/*spgM*+/*rpfF*+ were mostly strong biofilm producers. Among the antibiotic-resistance genes, the *Smqnr, L1, and sul1* had the highest prevalence (76.59%, 72.34%, and 64.89), respectively. Antimicrobial susceptibility evaluation showed 1.06%, 3.19%, and 6.3% resistance to minocycline, TMP-SMX, and levofloxacin.

**Conclusion:**

The results of the current study demonstrated that *S. maltophilia* isolates differ in biofilm-forming ability. Moreover, *smf*-1, *rmlA*, and *spgM* genes were presented in all strong biofilm producers. Although the overall resistance rate to the evaluated antibiotics was high, there was no statistically significant relation between antibiotic resistance and the type of biofilm.

## 1. Introduction

*Stenotrophomonas maltophilia* (*S. maltophilia*), an opportunistic, Gram-negative pathogen, can cause a wide variety of infections, including pneumonia, bacteremia, sepsis, meningitis, endocarditis, urinary tract infections, skin and soft tissue infections, and endophthalmitis [1]. One of the fundamental problems in the diagnosis of *S. maltophilia* is the high similarity with other nonfermenting Gram-negative bacteria, which has created many challenges in the diagnosis, treatment, and the prevalence of antibiotic resistance around the world. To overcome the problems associated with definitive identification, molecular methods have been developed [2]. It is mainly found in hospitalized patients, especially among the immunosuppressed and immunocompromised and those with medical implants, receiving broad-spectrum antibiotics, or suffering from cystic fibrosis [3]. Various virulence factors could be involved in the pathogenesis of *S. maltophilia*, including protease StmPr1, exopolysaccharides, lipopolysaccharides, siderophores, and the ability to form the biofilm [4]. Through biofilm formation, these bacteria could grow on biotic and abiotic surfaces, acting as a source of various infections and associated with more than 60% of acquired nosocomial infections [5]. Biofilms play a crucial role in the persistence of *S. maltophilia*healthcare-associated infections, especially in patients with implanted medical devices and cystic fibrosis patients [6]. Furthermore, bacterial biofilms increase the survival rate of bacteria under harsh conditions in the host imposed by the immune system or antibiotic therapies. The occurrence of antibiotic resistance due to biofilm formation makes treatment options more difficult [7]. It should be noted that *S. maltophilia* is usually resistant to several antibiotics because it confers various mechanisms of drug resistance, such as decreased permeability, production of beta-lactamase and carbapenemase enzymes, aminoglycoside-modifying enzymes, and MDR efflux pumps [8, 9]. At least two chromosomally mediated inducible beta-lactamases, *L1* and *L2*, are produced by *S. maltophilia*. An ampR-class A beta-lactamase module comprises the nearby ampR gene and gene *L2*, which encodes a class A beta-lactamase. *L1* is a class B beta-lactamase with no nearby regulatory genes resembling ampR [10]. The presence of *L1* contributes to hydrolyzing the carbapenems and resistance to clavulanic acid, cephalosporins, and penicillins. *L2* confers resistance to cephalosporins, penicillins, and aztreonam. *Smqnr* causes intrinsic resistance to quinolones [11]. Generally, this organism usually is susceptible to fluoroquinolones, minocycline, and ceftazidime [12]. Several studies have reported that trimethoprim-sulfamethoxazole (TMP-SMX) remains the drug of first choice for treating *S. maltophilia* infections. However, resistance to this antibiotic has been attributed to the presence of *sul1, sul2,* and *dfrA* genes [13]. Biofilms hinder the penetration of antibiotics; therefore, therapies may be faced with failure [14]. Accordingly, investigating the genes involved in biofilm formation and their role in the degree of biofilms has great importance. It can be helpful in the identification of strong biofilm producer isolates and adopting treatment strategies. So, the purpose of the present study was to evaluate antibiotic-susceptibility patterns, biofilm-forming capacity, antibiotic resistance genes, biofilm formation-associated genes, and the relationship between these genes with the degree of biofilm-formation capacity in *S. maltophilia* isolates.

## 2. Materials and Methods

### 2.1. Sample Collection

In this cross-sectional study, conducted from December 2021 to August 2022, 94 different clinical isolates of *S. maltophilia* were recovered from patients admitted to four tertiary-care hospitals in Iran (Tehran, Mashhad, Shiraz, and Qazvin). This study was approved by the Ethics Committee of Shahed University “IR.SHAHED.REC.1400.175.” The samples were identified using conventional microbiological and biochemical methods, including catalase and oxidase tests, DNase, urease, and reactions in media such as triple sugar iron (TSI) agar (Merck, Germany), Simmons citrate agar (Merck, Germany), and sulfide indole motility (SIM) (Merck, Germany) [15]. Stock cultures were stored in a tryptone soy broth (TSB) medium (Merck, Germany) containing 20% glycerol at −80°C until analysis. *Escherichia coli (E. coli)* ATCC 25922 and *S. maltophilia* ATCC 13637 were quality control strains.

### 2.2. DNA Extraction and Molecular Identification

All confirmed clinical isolates of S. *maltophilia* were cultivated on blood agar medium (Merck, Germany) and incubated at 37°C overnight. DNA was then extracted from colonies using the DNall Plus Kit (ROJE Co., Iran) according to the manufacturer's protocol. The total DNA concentration and purity were checked using the NanoDrop (WPA Biowave II Nanospectrophotometer, USA).

Finally, clinical isolates were confirmed based on the PCR amplification of the 23S rRNA gene and sequencing. PCR was conducted in a final volume of 25 *μ*l containing three *μ*l of template DNA, 12.5 *μ*l of Master Mix (2X) (ROJE, Iran), 1 *μ*l of each primer (10 pmol), and 7.5 *μ*l of sterile distilled water. More details about the primers and PCR conditions are given in Table 1.

### 2.3. Molecular Detection of Biofilm Formation and Antibiotic-Resistance Genes

The presence of biofilm-formation genes, including *smf*-1, *rmlA*, *spgM*, and *rpfF*, along with the antibiotic-resistance genes, including *L1*, *L2*, *sul1*, *sul2*, *sul3*, *dfrA13*, and *Smqnr*, was assessed by PCR using the primers and conditions indicated in Table 1. Amplifications were carried out in a thermocycler (Eppendorf, Master Cycler Gradient, Germany). PCR products were analyzed by electrophoresis on 1.5% agarose gel in TBE buffer, stained with DNA-safe stain (SinaClon-Iran), and then visualization under the UV transilluminator.

### 2.4. Biofilm-Formation Assay

Microtitre plate assay method was used to investigate biofilm-formation capacity (strong, moderate, or weak) in *S. maltophilia* isolates, as described previously [21]. Briefly, an overnight culture of *S. maltophilia* in TSB with an optical density (OD) at OD600 = 0.1 (10^8^ CFU/ml) was prepared; then, 200 *μ*l of suspension was inoculated into a sterile 96-well flat-bottomed polystyrene microplate (SPL, Korea). Negative control wells contained fresh TSB media, and all experiments were triplicate. The microplates were incubated aerobically for 24 h at 37°C. After incubation, wells were washed three times with 250 *μ*l of the phosphate-buffered saline (PBS) (pH 7.2). The adherent biofilms were fixed with 250 *μ*l of methanol; after 15 min, the wells were discarded and dried at room temperature and stained for 15 min with 200 *μ*l of 1% crystal violet. Each well was rinsed three times with PBS to remove extra dye and then dried. In the end, biofilm samples were resolubilized with 200 *μ*l of acetic acid (33%) for 15 min, and the OD of each well was measured by 490 nm using a microplate reader (Elx808, BioTek, USA). The biofilm-formation levels were categorized into four groups according to the following criteria: no biofilm producer (OD_t_ ≤ OD_c_), weak-biofilm producer (OD_c_ *<* OD_t_ < 2 × OD_c_), moderate biofilm producer (2 × OD_c_ < OD_t_ < 4 × OD_c_), and strong biofilm producer (OD_t_ ≥ 4 × OD_c_) [22].

### 2.5. Antimicrobial Susceptibility Testing

Antimicrobial susceptibility testing for clinical isolates was determined by the Kirby–Bauer disc diffusion method on Mueller–Hinton agar plates at 37°C for 20–24 hours, according to the guidelines of the Clinical and Laboratory Standards Institute (CLSI 2022). The McFarland 0.5 standard was used to standardize the inoculum density for susceptibility tests [23]. The antibiotic discs used in the current study included TMP-SMX (1.25/23.75 *μ*g), levofloxacin (5 *μ*g), and minocycline (30 *μ*g) (Liofilchem, Italy). *E. coli* ATCC 25922 was used as the control to check the accuracy of the susceptibility testing.

### 2.6. Statistical Analysis

Data analysis was performed using SPSS version 25.0 (IBM Corp., Armonk, NY, USA). The chi-square (*χ*^2^) test was conducted to determine the association between the two categorical variables. *P* value ≤0.05 was considered statistically significant.

## 3. Results

### 3.1. Patients and Samples

**Figure 1:**
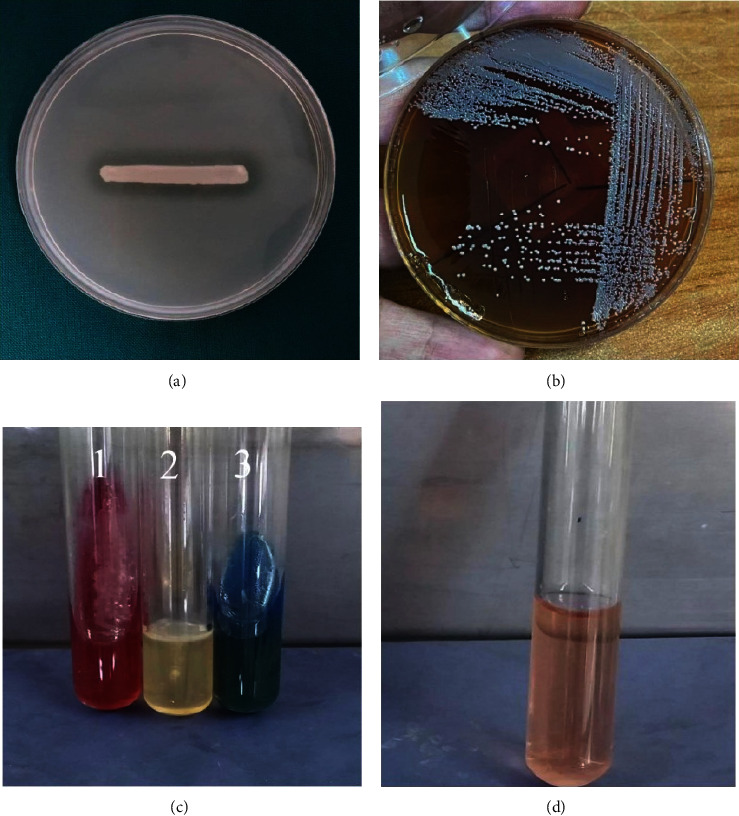
Conventional microbiological and biochemical methods for identification of *S. maltophilia*: (a) positive reaction for DNase. (b) Growth on blood agar medium after 24 hours of incubation at 37°C. (c) 1: nonfermenting organisms in TSI medium; 2: motile in SIM medium; 3: growth on Simmons citrate agar. (d) Negative urease test.

**Figure 2:**
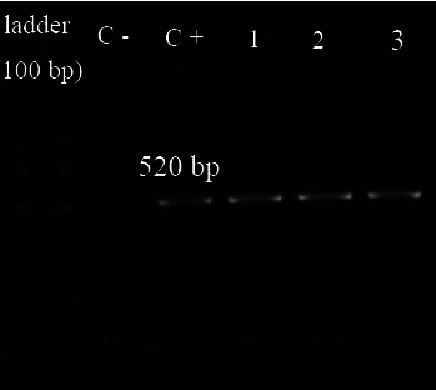
Electrophoresis gel of the PCR products of *23S rRNA* gene. C+: positive control, C−: negative control, and lanes 1–3: PCR-positive isolates.

Ninety-four clinical isolates of *S. maltophilia* were collected during the study period. The results of tests for initial identifications of clinical isolates are demonstrated in Figure 1. Moreover, the identity of all of the isolates was confirmed by amplifying the 23S rRNA gene by molecular PCR method; and in all of the clinical isolates, the PCR product was 520 base pairs long (Figure 2). The isolates were recovered from 57 (60.64%) males and 37 (39.36%) females. The patient's ages ranged from 7 months to 95 years. Isolate sources included blood (*n* = 86), urine (*n* = 3), tracheal secretion (*n* = 2), sputum (*n* = 2), and wound (*n* = 1) (Table 2). The majority of isolates were collected from patients hospitalized in the ICU (*n* = 40), followed by surgery (*n* = 19), internal (*n* = 17), emergency ward (*n* = 12), pediatric ward [5], and coronary care unit (CCU) (*n* = 1).

### 3.2. Biofilm Formation

#### 3.2.1. Phenotypes

The current study evaluated the ability of *S. maltophilia* isolates to form biofilms on polystyrene using the microtiter plate method. The phenotypic results demonstrated that the biofilm-formation rate was 98.93%, being distributed as follows: 18 isolates (19.35%) produced strong biofilm, 56 isolates (60.22%) produced moderate biofilm, and 19 isolates (20.43%) produced weak biofilm. Only one isolate produced no biofilm.

#### 3.2.2. Genotypes

**Figure 3:**
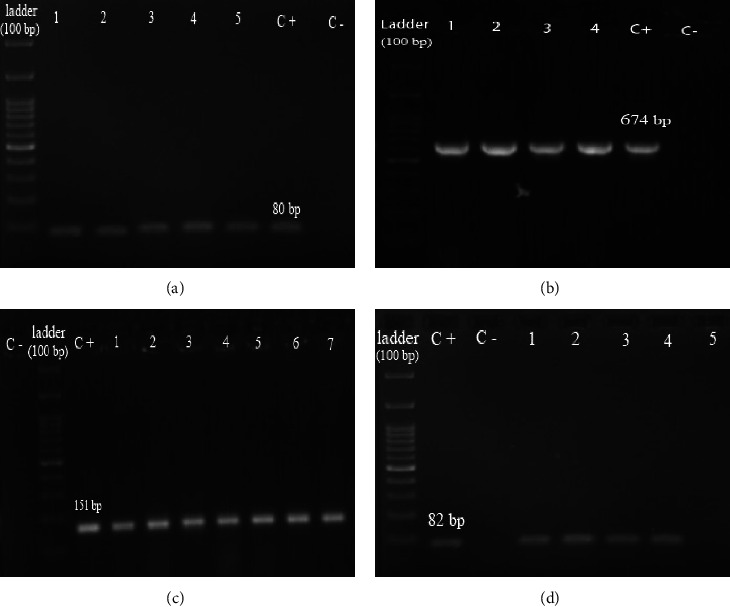
The gel electrophoresis images of PCR product of biofilm-forming genes, C+: positive control, and C−: negative control. (a) *spgM* (lanes 1–5: PCR-positive isolates). (b) *smf-1* (lanes 1–4: PCR-positive isolates). (c) *rpfF* (lanes 1–7: PCR-positive isolates). (d) *rmlA* (lanes 1–4: PCR-positive isolates and lane 5: PCR-negative isolate).

*(1) Investigation of Biofilm-Formation Genes and Their Relation with Biofilm-Forming Ability*. The frequency of biofilm-formation genes among 94 clinical *S. maltophilia* isolates was generally as high as 100%, 97.88%, 96.80%, and 75.53% for *spgM, rmlA, smf*-1, and *rpfF* genes, respectively (Figure 3). Five genotypes were observed to have a wide prevalence range (from 1.06% to 73.40%). The predominant genotype was *smf*-1+/*rmlA*+/*spgM*+/*rpfF*+ (73.40%), followed by *smf*-1+/*rmlA*+/*spgM*+/*rpfF*- (21.28%). Among investigated isolates, 73.4% carried all four genes mentioned above. The *rmlA* gene was presented in all moderate and strong biofilm producers and 94.73% of weak-biofilm producers. The presence of the *spgM* gene was confirmed among all isolates. Mainly isolates with the genotype *smf*-1+/*rmlA*+/*spgM*+/*rpfF*+ (77.78%) and *smf*-1+/*rmlA*+/*spgM*+/*rpfF*- (22.22%) formed strong biofilm (Table 3).

### 3.3. Investigation of Antibiotic-Resistance Genes and Their Relation with the Degree of Biofilms

**Figure 4:**
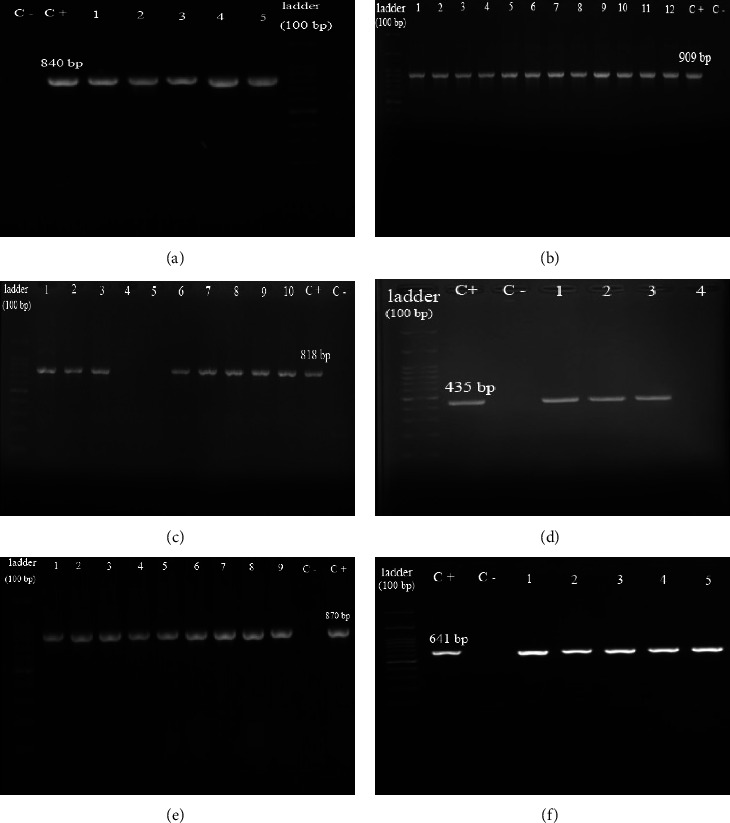
The gel electrophoresis images of PCR product of antibiotic-resistance genes, C+: positive control, and C−: negative control. (a) *sul1* (lanes 1–5: PCR-positive isolates). (b) *L1* (lanes 1–12: PCR-positive isolates). (c) *Smqnr* (lanes 1–3: PCR-positive isolates, lanes 6–10: PCR-negative isolate). (d) *sul2* (lanes 1–3: PCR-positive isolates and lane 4: PCR-negative isolate). (e) *L2* (lanes 1–9: PCR-positive isolates). (f) *dfrA13* (lanes 1–5: PCR-positive isolates).

The most and least genes present in *S. maltophilia* isolates were *Smqnr* (76.59%) and *dfrA13* (5.32%), respectively (Table 4). The *sul3* gene was not observed in any of the clinical specimens. The most antibiotic-resistance genes in strong and moderate biofilms strains were *sul1* and *dfrA13* genes, respectively (Table 4). The gel electrophoresis image of PCR products is demonstrated in Figure 4.

### 3.4. Antibiotic-Susceptibility Profile

*S. maltophilia* isolates showed the highest resistance to levofloxacin. Resistance to levofloxacin and TMP-SMX was 6.3% and 3.19%, respectively. Only one isolate was resistant to minocycline (Table 5).

## 4. Discussion

*S. maltophilia* is an opportunistic pathogen whose infection-crude mortality rate is about 14% to 69% of patients [24]. The ability to produce biofilms on biotic and abiotic surfaces, including indwelling medical devices, leads to an increasing prevalence of drug resistance in these Gram-negative bacteria [25]. This study demonstrates the genes involved in biofilm formation and antibiotic resistance and the correlation of these genes with the degree of biofilm-formation capacity in clinical isolates. Similar to the findings of this study, Bostanghadiri et al. in Iran reported that 98.7% and 95.7% of *S. maltophilia* isolates were biofilm producers, respectively, with a variable capacity of biofilm formation [11, 16]. Pompilio et al. reported that the frequency of biofilm formation of *S. maltophilia* in Italy was 88.2% [26]. In a study by Gallo et al. in Brazil, 96.7% of *S. maltophilia* were biofilm producers [27]. Notably, in a study conducted in Egypt, all *S. maltophilia* isolates were biofilm producers [28]. These results showed that *S. maltophilia* mostly have a high biofilm-producing ability, which can be important in the severity of virulence and antibiotic resistance of isolates. The isolates were classified into four categories based on the OD values using a microtiter plate assay to quantify biofilms. The estimated OD490 range (0.106–3.751) showed a 35-fold difference between the weakest and strongest biofilm producers. Our findings revealed that 19.15%, 59.58%, 20.21%, and 1.06% of isolates were strong, moderate, weak, and none-biofilm producers, respectively. Consistently, in various studies that investigated biofilm-forming ability in *S. maltophilia* isolates, a significant variation was reported [5, 16, 29]. These variations may be related to the presence of various biofilm-forming genes. In our research, we investigated the prevalence of biofilm-forming genes among *S. maltophilia* strains. We observed that the frequency of *spgM*, *rmlA*, *smf-*1, and *rpfF* genes was 100%, 96.80%, 97.88, and 75.53, respectively. Since the formation of biofilms involved in antimicrobial resistance as well as immune evasion, the average ability of biofilm formation of *S. maltophilia* isolates has been investigated by various studies, including strong biofilm producers with a frequency of 10% to 98.4%, moderate biofilm producers with the frequency of 21.3% to 46.6%, and weak-biofilm producers with a frequency of 16% to 36.6% [30]. *spgM* encodes a bifunctional enzyme with both phosphoglucomutase and phosphomannomutase activities [5]. Different reports revealed that the *spgM* gene could play an important role in biofilm development, and its presence was significantly associated with producing strong biofilm in *S. maltophilia* isolates [16, 29]. Bostanghadiri et al. [31] and Duan et al. [32] reported that all *S. maltophilia* isolates harbor the *spgM* gene, similar to our study. *The rpfF* gene plays a significant role in producing the signal molecule known as a diffusible signal factor, a highly conserved quorum sensing signal in Gram-negative bacteria [33]. Madi et al. [34] and Zhuo et al. [5] reported that the lowest frequency of biofilm-forming genes belonged to *rpfF*, similar to the current study. These results demonstrated that geographical variation does not affect the prevalence of this gene in *S. maltophilia* isolates. *smf*-1 involves adhesion to various surfaces and the initial stages of biofilm formation. In China and Egypt, the frequency of *the smf*-1 gene in *S. maltophilia* isolates was as high as 100% and 90%, respectively [28, 32]. Unlike our study, a low level of *the smf*-1 gene was seen in the study conducted by Mohagheghzadeh et al. [35]. This difference might be due to time of study. The *rmlA* encodes a glucose-1-phosphate thymidyl transferase. These products are essential for the biosynthesis of lipopolysaccharide (LPS) O-antigen, which is involved in biofilm formation and twitching motility [36]. In previous studies, the *rmlA* gene had a high frequency in *S. maltophilia* isolates [5, 16, 34]. Our findings demonstrated increased resistance to several antibiotics commonly used in *S. maltophilia* infections in Iran [11, 31, 37, 38]. The antimicrobial susceptibility profile showed that 1.06%, 3.19%, and 6.3% of isolates were resistant to minocycline, TMP-SMX, and levofloxacin, respectively. These results emphasize the urgent need to address the inappropriate use and prescription of antibiotics and implement robust control policies to limit the dissemination of resistant *S. maltophilia* strains and protect public health. Gajdács and Urbán reported that 12.1% of *S. maltophilia* isolates were TMP-SMX-resistant, while 8.99% were resistant to levofloxacin [39]. Compared to our study, the resistance to TMP-SMX was almost four times higher, possibly due to the difference in the geographical area, type of antimicrobial susceptibility test, and the year of study. TMP-SMX is the primary antimicrobial drug of choice for treating *S. maltophilia* infections although, in recent years, increasing rates of resistance from 2.3% to 77% were reported [30]. Based on a systematic review study, fluoroquinolones demonstrate comparable effects on the mortality of *S. maltophilia* infection compared with TMP-SMX, supporting the use of fluoroquinolones (mainly levofloxacin) in *S. maltophilia* infections [40]. However, a high resistance level is also increasingly mentioned [26]. The increase in antimicrobial resistance continues to be a global health crisis [41]. One of the mechanisms involved in resistance to various antibiotics in *S. maltophilia* isolates is the presence of antibiotic-resistance genes. Our research investigated the prevalence of antibiotic-resistance genes, including *L1*, *L2*, *Smqnr*, *sul1*, and *sul2*, among *S. maltophilia* strains in patients referred to different hospitals in Iran. Our findings revealed that 64.89%, 32.97%, and 76.59 strains harbored the *sul1*, *sul2*, and *Smqnr* genes, respectively, in accordance with those obtained by Ebrahim-Saraie [42]. This result indicated high prevalence of antibiotic-resistance genes in Iran, and this issue can be alarming for spread of resistant isolates in society. In a study conducted by Yinsai et al. in Thailand, only 6% and 2% of *S. maltophilia* strains were positive for the *sul1* and *sul2* genes, respectively, which contradict our results [43]. These variations in prevalence could be attributed to differences in population, time of study, clinical samples, and geographical factors. In the current study, the presence of *dfrA13* was reported in clinical isolates of *S. maltophilia* for the first time in Iran, and the frequency of this gene was 5.32%. Similar to other studies, *sul3* was not detected in clinical isolates of *S. maltophilia* [44, 45]. This newest sulfonamide-resistance gene is usually located on plasmid and especially isolated in nonclinical specimens including water, soil, sewage loving animals, and animal farm [18]. More clinical studies are required to figure out better. Similarly, various studies revealed that most *S. maltophilia* isolates were recovered from blood and mainly isolated from males [11]. The advent of bloodstream infections caused by *S. maltophilia* and its associated complications has significantly increased in recent years, with the range of mortality rate as 30–51% [46]. This rise can be attributed to the excessive and frequent consumption of available antibiotics. Notably, our study found that all resistant isolates were obtained from blood samples of patients with bacteremia in different regions of Iran. *S. maltophilia* was mainly isolated from the ICU, which is similar to the study by Ibn Saied et al. [47]. Considering that most patients who are hospitalized in the ICU are immunocompromised, more attention should be given to the accurate identification and proper treatment of this opportunistic bacterium. This study has some limitations that can be mentioned as follows: First, almost all *S. maltophilia* isolates were biofilm producers; therefore, we could not compare various genes in clinical specimens with/without biofilm. Second, the other mentioned antibiotics based on ETEST according to CLSI guidelines were not reported in the current study; therefore, we could not demonstrate a comprehensive antimicrobial drug susceptibility profile in *S. maltophilia* isolates. Third, we could not present the phylogenetic relationship of antibiotic-resistant and strong biofilm-producer isolates in the current study due to the lack of molecular typing.

## 5. Conclusion

The ability to form biofilms in *S. maltophilia* makes antibiotics ineffective and rapidly growing drug resistance of pathogenic bacteria. It is demonstrated that although the capacity to form biofilm in clinical isolates of *S. maltophilia* was highly conserved, there are multiple phenotypic variations among them. Moreover, the *spgM* gene was presented in biofilm- and nonbiofilm-producing isolates. Furthermore, the most antibiotic-resistance gene in the two mentioned groups was *Smqnr*.

## Figures and Tables

**Table 1:** Primers used in this study.

Primers	Sequences (5′–3′)	Cycles	Initial denaturation	Cycling	Final extension	Length (bp)	References
*23S rRNA*	F: CAGCCTGCGAAAAGTA R: TTAAGCTTGCCACGAAC	30	5 min, 95°C	30 s, 94°C; 45 s, 54°C; 1 min, 72°C	7 min, 72°C	520	[16]

*smf-1*	F: GGAAGGTATGTCCGAGTCCG R: GCGGGTACGGCTACGATCAGTT	30	5 min, 95°C	45 s, 94°C; 45 s, 62°C; 45 s, 72°C	7 min, 72°C	674	[16]

*rmlA*	F: GCAAGGTCATCGACCTGG R: TTGCCGTCGTAGAAGTACAGG	30	5 min, 95°C	20 s, 94°C; 20 s, 60°C; 30 s, 72°C	10 min, 72°C	82	[16]

*spgM*	F: GCTTCATCGAGGGCTACTACC R: ATGCACGATCTTGCCGC	30	5 min, 95°C	20 s, 94°C; 20 s, 60°C; 30 s, 72°C	10 min, 72°C	80	[16]

*rpfF*	F: CTGGTCGACATCGTGGTG R: TGATCCGCATCATTTCATGC	36	5 min, 94°C	45 s, 94°C; 45 s, 59°C; 45 s, 72°C	5 min, 72°C	151	[16]

*L1*	F: AGCCGTTAAAATTAAGCCC R: CTTGATTGAAGGGTTGGGCG	36	5 min, 94°C	45 s, 94°C; 45 s, 56°C; 45 s, 72°C	5 min, 72°C	909	[11]

*L2*	F: CGATTCCTGC AGTTCAGT R: CGGTTACCTC ATCCGATC	36	5 min, 94°C	30 s, 94°C; 30 s, 55°C; 30 s, 72°C	5 min, 72°C	870	[17]

*sul1*	F: ATGGTGACGGTGTTCGGCATTCTGA R: CTAGGCATGATCTAACCCTCGGTC	36	5 min, 94°C	45 s, 94°C; 45 s, 62°C; 45 s, 72°C	5 min, 72°C	840	[11]

*sul2*	F: GAAGCGCAGCCGCAATTCAT R: CCTGTTTCGTCCGACACAGA	36	5 min, 94°C	45 s, 94°C; 45 s, 60°C; 45 s, 72°C	5 min, 72°C	435	[11]

*sul3*	F: GAGCAAGATTTTTGGAATCG R: CATCTGCAGCTAACCTAGGGCTTTGGA	30	5 min, 94°C	60 s, 94°C; 60 s, 55°C; 1 min, 72°C	7 min, 72°C	799	[18]

*Smqnr*	F: ACACAGAACGGCTGGACTGC R: TTCAACGACGTGGAGCTGT	36	5 min, 94°C	45 s, 94°C; 45 s, 59°C; 45 s, 72°C	5 min, 72°C	818	[19]

*dfrA13*	F: CTCATCTGCTGGCTATCTCA R: GAAACTATCACTAATGGCAGC	36	5 min, 94°C	45 s, 94°C; 45 s, 59°C; 45 s, 72°C	5 min, 72°C	641	[20]

**Table 2:** Clinical sources of *S. maltophilia* and their prevalence in different biofilm degrees.

Clinical sources (n (%))	Degree of biofilm (*n* (%))			
	Strong (%)	Moderate (%)	Weak (%)	None (%)
Blood 86 (91.49)	16 (18.61)	53 (61.62)	16 (18.61)	1 (1.16)
Urine 3 (3.19)	0.0 (0)	2 (66.7)	1 (33.3)	0.0 (0)
Tracheal secretion 2 (2.13)	1 (50)	0.0 (0)	1 (50)	0.0 (0)
Sputum 2 (2.13)	0.0 (0)	1 (50)	1 (50)	0.0 (0)
Wound 1 1 (1.06)	1 (100)	0.0 (0)	0.0 (0)	0.0 (0)
Total 94 (100)	18 (19.15)	56 (59.58)	19 (20.21)	1 (1.06)

**Table 3:** Association between biofilm-forming genes in different categories of biofilm-forming *S. maltophilia* isolates (*n* = 94).

Biofilm-formation genes	No. (%) of isolates	Degree of biofilm *n* (%)				
		Strong (*N* = 18)	Moderate (*N* = 56)	Weak (*N* = 19)	None (*N* = 1)	*P* value
*smf*-1	91 (96.80)	18 (19.78)	54 (59.34)	18 (19.78)	1 (1.10)	*P* > 0.05
*rmlA*	92 (97.88)	18 (19.56)	56 (60.88)	18 (19.56)	0.0 (0)	*P* ≤ 0.05
*spgM*	94 (100)	18 (19.15)	56 (59.58)	19 (20.21)	1 (1.06)	*P* > 0.05
*rpfF*	71 (75.53)	14 (19.72)	42 (59.15)	14 (19.72)	1 (1.41)	*P* > 0.05

**Table 4:** Association between antibiotic-resistance genes in different categories of biofilm-forming *S. maltophilia* isolates (*n* = 94).

Antibiotic-resistance genes	No. (%) of isolates	Degree of biofilm *n* (%)				
		Strong (*N* = 18)	Moderate (*N* = 56)	Weak (*N* = 19)	None (*N* = 1)	*P* value
*L1*	68 (72.34)	15 (22.06)	38 (55.88)	14 (20.59)	1 (1.47)	*P* > 0.05
*L2*	48 (51.06)	8 (16.67)	29 (60.42)	10 (20.83)	1 (2.08)	*P* > 0.05
*sul1*	61 (64.89)	11 (18.03)	35 (57.38)	15 (24.59)	0.0 (0)	*P* > 0.05
*sul2*	31 (32.98)	5 (16.13)	15 (48.39)	11 (35.48)	0.0 (0)	*P* > 0.05
*Smqnr*	72 (76.59)	16 (22.22)	40 (55.56)	15 (20.83)	1 (1.39)	*P* > 0.05
*dfrA13*	5 (5.32)	0.0 (0)	4 (80)	1 (20)	0.0 (0)	*P* > 0.05

**Table 5:** Antibiotic susceptibility of the *S. maltophilia* isolates (*n* = 94).

Antimicrobial agents	Disc diffusion number (%)		
	S	I	R
Levofloxacin	88 (93.62)	0.0 (0)	6 (6.38)
Minocycline	90 (95.75)	3 (3.19)	1 (1.06)
TMP-SMX	91 (96.81)	0.0 (0)	3 (3.19)

TMP-SMX: trimethoprim-sulfamethoxazole; S: susceptible; I: intermediate; R: resistant.

## Data Availability

The data used to support the findings of this study are available from the corresponding author upon reasonable request.
